# Establishing Quality and Outcome Measures for Recovery Housing: A Tiered Approach Supporting Service Evolution

**DOI:** 10.1007/s10597-023-01219-6

**Published:** 2024-01-25

**Authors:** Robin A. Thompson, David Johnson, Madison Ashworth, Milena Stott

**Affiliations:** Fletcher Group, Inc., 601 Meyers Baker Road, Suite 238, London, KY 40741 USA

**Keywords:** Recovery housing, Outcome measures, Substance use disorder, Recovery

## Abstract

With over one-hundred thousand drug overdose deaths in 2021, substance use disorder (SUD) is a public health crisis in the United States. Medical stabilization has been the predominant focus of SUD interventions despite low levels of retention. Consequently, national quality measures for SUD care outside the clinical continuity of care are limited. The expansion of recovery support services addressing social drivers of health outside clinical settings is needed. The current SUD quality measures are not applicable nor feasible for recovery support service providers with limited resource capacities, like the estimated 17,900 recovery housing providers nationwide. Despite widespread support for recovery housing and its documented effectiveness, no universal set of measures has been developed for widespread adoption. In this brief, a matrix of quality measures are proposed to meet the needs of recovery housing providers with various capacities to support service evolution and improve equitable SUD treatment and recovery care.

## Current National Efforts – Substance Use Disorder Outcomes

With over one-hundred thousand lives lost due to drug overdose in 2021, substance use disorder (SUD), is a public health crisis in the United States (U.S.) (Ahmad et al., 2021). This worsening crisis presents a multifaceted challenge due to the combination of risk factors contributing to SUD and other ailments often co-occurring with SUD (Ogden et al., 2022; Stone et al., 2012). Further complicating SUD is the pervasive social stigma, which often impacts treatment seeking as well as access to housing and employment to support the recovery process (Zwick et al., 2020). Access to SUD care is a nationwide issue, but disproportionate disparities are continuously witnessed among rural residents due to hospital closures, lack of providers, and limited internet access (United States Government Accountability Office, 2023). This issue was recently highlighted in 2030 Rural Healthy People, as “healthcare quality and access”, and “addiction” were identified as top priorities by rural health stakeholders (Kassabian et al., 2023). These barriers compound the already challenging task of recovering from SUD. Individuals impacted by SUD often attempt recovery five times on average before resolving their SUD (Kelly et al., 2019). SUD recovery interventions include outpatient, intensive outpatient, and 30–90-day inpatient detoxification and treatment programs, prescription medications (e.g., buprenorphine, naltrexone, Subutex, etc.) while social recovery models include mutual aid (e.g., 12-step, AA/NA), peer-support, and recovery housing (RH).

In 2022, the Substance Abuse and Mental Health Services Administration’s (SAMHSA) interim strategic plan described a vision for promoting recovery that includes the development of SUD recovery facilitating environments, such as RH (Substance Abuse and Mental Health Services Administration, 2022). Further, they articulated their commitment to data and evidence in determining the impact of programs on substance use and mental health conditions. In the area of substance use, the most frequently used quality measures employed include process measures promoted by the Center for Medicare and Medicaid Services (CMS) as defined by the National Committee for Quality Assurance (NCQA) in collaboration with the National Quality Forum (NQF). In 2020, five quality measures were identified for substance use (Centers for Medicare and Medicaid Services, Medicaid and CHIP, 2023):

1. Initiation and engagement of alcohol and other drug abuse (AOD) or dependence treatment;
2. Use of opioids at high dosage in persons without cancer;
3. Concurrent use of opioids and benzodiazepines;
4. Use of pharmacotherapy for opioid use disorder; and
5. Follow-up after emergency visit for alcohol and other drug abuse or dependence.

These five SUD process measures are limited in their focus on SUD treatment, which are broad in nature and primarily focused on outpatient services. Measures two and three are primarily focused on pain management and not directly related to SUD treatment while the fifth, is limited to assessment post-emergency department visit. Other commonly used quality outcome measures are SAMHSA’s National Outcome Measures, developed in response to the Government Performance and Results Act (GPRA), which is a comprehensive system covering ten domains with standardized data collection tools and prescribed collection points: baseline, three, six and twelve months; these measures are required for recipients for Block Grant and State Opioid Response funds (Substance Abuse and Mental Health Services Administration, n.d.-b).

The evolving need to evaluate outcomes is reflected by the move to value-based payment models. In 2010, CMS developed an innovation center to inform development of a value-based system that would reduce costs and enhance quality of care (Brooks-LaSure et al., 2021). This innovation center involves the development, testing, and evaluation of new payment and service delivery models with the following objectives: drive accountable care, advance health equity, support innovation, address affordability, and partner to achieve system transformation (Centers for Medicare and Medicaid Services, 2021). To achieve equitable, accountable care for individuals with SUD, innovative strategies outside the standard healthcare system will be required as it has been estimated that 80% of health outcomes are impacted by non-clinical social drivers of health (SDOH) (Elevance Health, n.d.; Manatt et al., 2019). Leveraging providers of recovery support services and supporting their evaluation capacities will enable resources to be allocated to those recovery support services that ultimately address the SDOH that impact SUD healthcare access. In this review, we discuss the current measures used to evaluate SUD and recovery support services, and the evaluation tool gaps for SUD recovery support service models. We then present an adapted continuity of care framework developed for the context of RH along with a protocol including proposed process and outcome indicators for four tiers depicting a service provider’s increasing capacities.

## The Need for Universal Substance Use Disorder Service Data Collection Measures

It is important to account for the full continuum of care including traditional clinical models of outpatient and inpatient services as well as social recovery service models when developing quality measures. Social recovery service models are diverse and include recovery community organizations, RH programs, supportive employment, and other mutual aid programs. These service models are critical to supporting individuals in or seeking SUD recovery as they address key SDOH factors. Thus, there is a need to develop standardized measures that can be universally adopted, are person centric, and reflect the range of programs and services addressing SUD.

The existing measures are limited in terms of their application by service context (i.e., clinically oriented for a healthcare setting), and thus tend to not be person centric, given an individual experiencing an SUD may receive care from healthcare and additional recovery support settings. Further, the length of the GRPA tool is burdensome for providers of other SUD recovery supports like RH, where there are limited resources to support data collection activities (Ashworth et al., 2022; Substance Abuse and Mental Health Services Administration, n.d.-a).

The Fletcher Group, serving as a Rural Center of Excellence with focus on Recovery, is working to define quality RH programs and services. Fletcher Group subject matter experts proposed a model that is flexible, and person and program centric. The model builds on previous research of transtheoretical models of outcomes informed care, and identifies options based on the size and resources of RH programs (Duncan & Reese, 2015; Guo et al., 2015; Kopta et al., 2015; Lambert, 2015; Lewis et al., 2015; Miller et al., 2015; Wampold, 2015). In developing and implementing value-based payment models for the healthcare system, as well as social recovery service support system, there are several pragmatic challenges to keep in mind. For example, models for home and community-based services often face feasibility and capacity issues relating to implementing value based payment models similar to those faced by providers of equally important RH models (Lipson et al., n.d.).

RH, defined by SAMHSA as “… safe, healthy, family-like substance-free living environments that support individuals in recovery from addiction”, represents an important recovery support service, with an estimated 17,900 nationwide serving over 275,000 people at any given time (Jason et al., 2020; Substance Abuse and Mental Health Services Administration, 2018). The National Alliance for Recovery Residences (NARR) defines four levels of RH (I-IV), that depict levels of increasing support via staffing and service intensity provided in a home (National Alliance for Recovery Residences, 2018). Level I homes are characterized as peer-run homes with no paid staff and limited services (peer led house meetings and peer support) provided in-house. Level IV, the highest, characterizes a home with peer specialists and professional staff and the provision of contracted medical services in-home (e.g., behavioral health counseling). Recovery houses are privately owned and operated, and vary considerably in terms of available resources, resident census, populations served, recovery pathways offered, and strength of the recovery ecosystem of the community in which they are located (*Recovery Ecosystem Index (REI)*, n.d.). In rural communities, where SUD and recovery support services may be limited, a recovery house may be a major resource for individuals with a SUD (*Recovery Ecosystem Index (REI)*, n.d.; United States Government Accountability Office, 2018). Due to the heterogeneity of these factors across homes nationwide and lack of standardized universally adopted measures, data collection efforts vary considerably.

The evidence for RH effectiveness is growing but challenges with an undetermined national capacity and service utilization have resulted in a limited number of rigorous studies (United States Government Accountability Office (GAO), 2018). A systematic review of recovery support services in the United States conducted by Kelly in 2021 indicated that a total of 10 quasi-experimental studies of RH have been conducted representing three original studies and that the scientific rigor on RH can be viewed as “moderate” (Kelly, 2017). In 2020, Mericle and colleagues were awarded a grant through the National Institute on Alcohol Abuse and Alcoholism at the National Institutes of Health to conduct the National Study of Treatment and Addiction Recovery Residences project, to develop a comprehensive database of the RH landscape (Mericle et al., 2022). This study, along with previously conducted research indicates that RH is promising, with documented positive impacts on recidivism, abstinence, reduction in drug overdose mortality, and employment (Chavarria et al., 2012; Jason et al., 2006, 2016; Polcin et al., 2010; Tuten et al., 2012, 2017). Recovery Kentucky, a RH model established by former Governor Ernie Fletcher, has documented 10 years of positive outcomes (Cole et al., n.d.). Despite the positive outcomes associated with RH that are reported in a limited number of studies that employed rigorous designs, there is a growing need to systematically assess the impacts of the various RH models which can be used to elevate these recovery support services from auxiliary, wrap-around services, to primary SUD services (American Society of Addiction Medicine (ASAM), n.d.).

SAMHSA, the United States Department of Housing and Urban Development, and Government Accountability Office have all published issue briefs related to RH in the past five years, yet there is limited government oversight and no universal set of measures for data collection in RH (United States Department of Housing and Urban Development, 2015; SAMHSA, 2018; GAO, 2018). Additionally, NARR has established widely recognized and adopted RH certification standards, yet these standards only address facility and operational issues and do not specify reporting of measures addressing resident outcomes (NARR, 2018). Funding opportunities specific to RH provided through states such as the Substance Abuse Prevention and Treatment Block grant, State Opioid Response Grants, or other foundation funding opportunities often requires the collection of a minimum set of performance or service utilization indicators like GPRA but due to the heterogeneity of reporting requirements, RH data collection varies significantly (Fletcher Group, Inc., 2023a; Substance Abuse and Mental Health Services Administration, 2023b; Substance Abuse and Mental Health Services Administration, n.d.). In a recent 2022 study of single state agencies (SSAs), among the 48 SSAs that participated, 71% indicated that their SSA captures demographic information and 50% indicated their SSA collects outcomes data on residents of state-supported RH (Fletcher Group, Inc., 2023b). Additionally, 83% of SSAs indicated that RH resident outcomes data being available was “important” or “very important”.

**Fig. 1:**
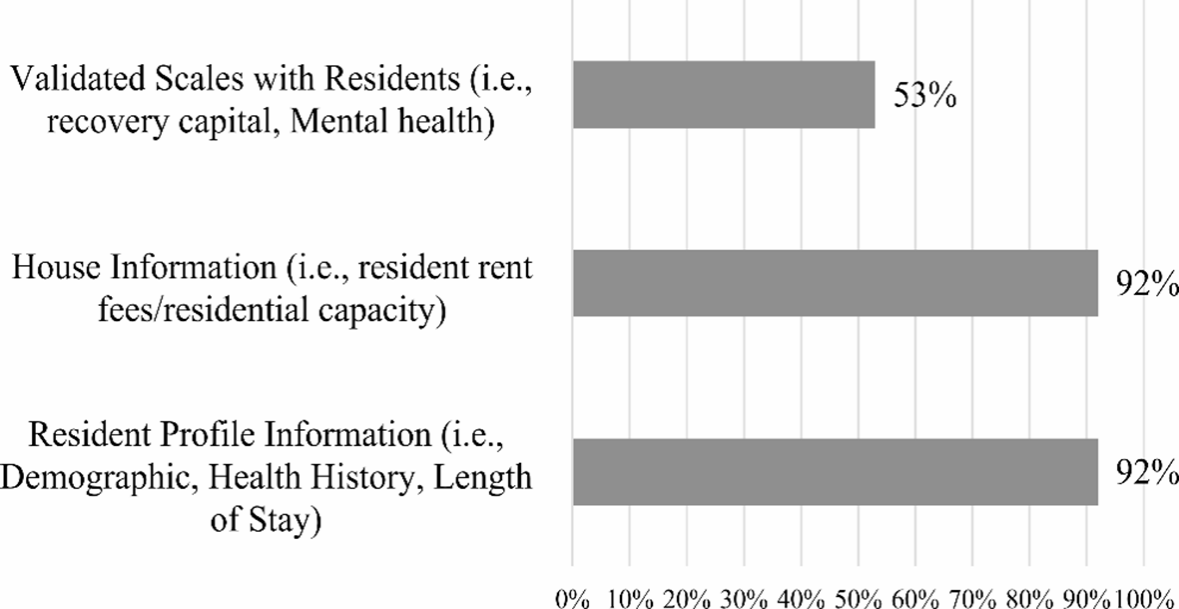
Partial sample of the recovery housing national capacity surveillance survey, data collection by data types (N = 36), Fletcher Group, United States, 2023

In March 2021, the Fletcher Group began conducting a RH national capacity surveillance survey to assess the capacity and service details of RH nationwide (Fletcher Group Inc., 2023a). Still underway, a total of 66 RH providers representing 144 homes located across 14 states (Arkansas, California, Georgia, Idaho, Iowa, Maryland, Michigan, Montana, Ohio, Oklahoma, Tennessee, Washington, West Virginia, and Wyoming) have provided responses to the survey. Most providers (61%) indicated collecting data on their house and/or residents. Among those 40 providers that indicated data collection, 92% reported collecting resident profile information (i.e., demographic, health history and length of stay) and house information (i.e., resident rent/fees, residential capacity) (Fig. 1). A smaller portion (53%) indicated collection using validated instruments to measure recovery-related progress such as recovery capital and mental health.

To support RH adoption of a recovery outcomes informed care approach, Best and colleagues developed the recovery capital (REC-CAP) tool (Härd et al., 2022). This comprehensive online platform assesses residents’ barriers and unmet meets in addition to resources that can be used to support recovery. It assesses recovery capital with a combination of tools including the Assessment of Recovery Capital (ARC), the Commitment to Sobriety Scale, the Social Support Scale, and the Recovery Group Participation Scale (RGPS) (Groshkova et al. 2011, 2013; Jetten et al., 2011; Kelly & Greene, 2014). Although this approach is comprehensive and evidence-based, REC-CAP may require a substantial time commitment as well as a level of technological literacy for adoption and utilization. RH classified as levels I and II, with smaller staffs and fewer resources, or homes lacking experience in outcomes data collection may find such a system too challenging. Early pilot data collected with REC-CAP from 2016 to 2019 with 823 residents from level II homes under a single nonprofit indicate high engagement with the baseline survey and a third of residents completing follow-up assessment at six months (Härd et al., 2022). Homes participating in this study also employed a recovery coach with the primary focus on completion of the REC-CAP with residents. This represents an ideal scenario for data collection support in RH that is not often the norm for most level II recovery homes not operating with state funding. REC-CAP may be efficacious in that ideal, high resource availability setting, but its effectiveness and feasibility are unclear when implemented in houses across the country lacking implementation resources. Further, the cost to implement the REC-CAP system may be prohibitive for some houses, unless they are a NARR-certified house in a state covering the cost (Best et al., 2023; Härd et al., 2022). In terms of additional developments in supporting quality improvement of SUD care, through a non-experimental outcomes pilot project with eight substance use treatment facilities, the National Association of Addiction Treatment Providers (NAATP) developed and published a toolkit in 2019 to disseminate best practices for conducting outcomes research in substance use treatment programs (Hirsh et al., 2019). A valuable resource and step to improve standardization of measures across SUD facilities, the toolkit has a strong emphasis on supporting SUD treatment facilities conducting research and is geared towards serving licensed and accredited NAATP providers, representing a higher level of care often provided by smaller RH providers.

Despite the strong recognition and support for RH at the federal and state levels, as well as positive evidence for RH, no universal set of measures developed with the feasibility to meet needs of RH operators of varying capacities has been developed. The provision of a universal set of measures that meet the needs of homes at all levels to support evaluation capacities is crucial for the evolution of this recovery support service and in particular, for meeting the following objectives: examination of (a) which types of RH models work best for various populations in terms of demographic, cultural considerations and geographic location, SUD severity, and risk factors; (b) care continuity with RH; (c) RH availability and quality service gaps, geographically (rural and non-rural) and demographically, for individuals in need; and (d) effectiveness of RH as a recovery support serviceto support alternative payment models that achieve healthcare system transformation to improve equitable SUD treatment and recovery care.

### A Simple Yet Impactful Process Measure

In the SUD medical model of care, higher continuity of care with medication for addiction treatment (MAT) has been found to be associated with better SUD outcomes (Timko et al., 2016). Thus, there has been substantial emphasis on research aimed at identifying the predictors associated with improved MAT retention rates and resulting SUD outcomes. Initiation, a Healthcare Data and Information Set (HEDIS) measure is defined by the NCQA as “Adolescents and adults who initiated treatment through an inpatient AOD admission, outpatient visit, intensive outpatient encounter or partial hospitalization, telehealth or MAT within 14 days of diagnosis” (National Committee for Quality Assurance (NCQA), n.d.). Engagement, also a HEDIS measure is defined as “Adolescents and adults who initiated treatment and had two or more additional AOD services or MAT within 34 days of the initiation visit” (National Committee for Quality Assurance (NCQA), n.d.). Retention is a measure defined by the NQF as the “percentage of adults aged 18 years and older with pharmacotherapy for opioid use disorder (OUD) who have at least 180 days of continuous treatment” (American Medical Association, 2019). Length of stay in RH has also been studied in relation to its impact on recovery outcomes, although research is limited. Work led by Jason et al. assessing length of stay in Oxford House model indicates that residents that stay for at least six months have improved outcomes in the areas of abstinence, employment, and self-efficacy (Jason et al., 2007, 2016).

This simple process measure of care continuity is one that can be translated to the social model of RH and is feasible for collection by RH of all levels given it only requires documentation of residents’ entry and exit date. In 2021, RH and measurement subject matter experts from the Fletcher Group developed a retention framework for the context of RH, modeled and adapted from the medical care continuity retention framework developed by NCQA and NQF for initiation, engagement, and retention noted above. RH initiation was defined as a stay of at least one week (7 days) up to 29 days; engagement defined as a stay of at least one month (30 days) up to 179 days; and retention defined as a stay of at least 6 months (180 days) or more (Table 1). Despite evidence behind a six-month stay, not all RH residents will require six months for stabilization. In other cases, entry into a RH may be a continuation of treatment started in an acute care setting and thus is building upon prior service initiation. Additionally, many RH programs offer an open-ended length of stay directed by residents (Polcin, 2009). Services offered in-house or referred out also differ significantly across RH programs, thus, it’s imperative that documentation of these nuances occurs. Care continuity as a process measure for all RH providers will enable a simple, low burden measure for documentation of effectiveness, regardless of RH program heterogeneities.

**Table 1:** Care continuity measures for SUD clinical treatment adapted for SUD recovery context, “care continuity framework for recovery housing”, 2023

Process Measure	Treatment (Clinical Setting)	Recovery (Recovery Housing)
Initiation	Adolescents and adults who initiated treatment through an inpatient alcohol or other drug (AOD) admission, outpatient visit, intensive outpatient encounter or partial hospitalization, telehealth, or MAT within 14 days of diagnosis.	Residency in recovery housing for at least 7 days.
Engagement	Adolescents and adults who initiated treatment and had two or more additional AOD services or MAT within 34 days of the initiation visit.	Residency in recovery housing for at least 30 days.
Retention	Percentage of adults aged 18 years and older with pharmacotherapy for opioid use disorder (OUD) who have at least 180 days of continuous treatment.	Residency in recovery housing for at least 180 days.

This adapted care continuity framework was then applied by Thompson et al. (2023) in an analysis of data from 566 RH residents over a 17-year period (2005–2022). In this analysis, they found that 4% left the home within the first week, 14% left between one week and one month, 38% stayed from 30 to 179 days, and 44% stayed for 180 days or more reflecting initial longitudinal evidence for RH engagement, a recovery support service for individuals in recovery from a SUD (Fletcher Group, Inc., 2023a).

Comparatively, data indicates that measures of care continuity with MAT are low. National data reported by the NCQA indicate that in 2021, among adults and adolescents (13 years or older) with a new episode of alcohol or drug dependence, between 33.1 and 44.2% met criteria for initiation with medical treatment depending upon payer, and 4.5–13.9% met criteria for engagement with medical treatment depending on payer (National Committee for Quality Assurance (NCQA), n.d.).

This care continuity framework, adapted for the RH context, reflects an initial process measure that can be collected by a single measure at intake and departure. This framework developing an understanding of engagement with RH as well as serve as an initial measure to identify trends associated with premature departure to support quality improvement efforts with RH programming. Further, this framework is generalizable and can be adopted by RH in varying geographic locations (rural and non-rural). Current national data reflecting low care continuity with MAT indicate innovative alternatives that address SDOH to support individuals in recovery from an SUD are needed, especially in rural areas where resources for SUD are limited. With widespread adoption by RH of this single process measure, evidence for care continuity with this recovery support service may yield promising alternative payment models to help build sustainability to ensure widespread SUD recovery care access for those in need.

## Data Collection Sets Supporting All House Levels

Feasibility is an important element to consider with data collection in the RH context. RH operators have varying levels of capacities, in-house resources, and are located within communities with recovery ecosystems of varying strengths (*Recovery Ecosystem Index (REI)*, n.d.). For RH operators that may not be collecting any information, any level of data collection may be burdensome especially if resources such as staff and technology are limited. To build evaluation capacities with all RH operators regardless of their resource capacities, a tiered approach with four levels of proposed measures for collection is provided representing a minimum to robust data set. As tiers increase from one to four, the measures collected in the preceding tiers are included. Proposed measures and collection timepoints are provided:

### Tier 1: Minimum

This tier covers measures that will begin to define which populations are using RH and their length of stay. There is little known about which populations currently utilize RH and where service access disparities may exist. Length of stay is a simple measure collected at intake and departure that represents continuity of care with RH. First, collecting length of stay may enable house staff to begin to assess average length of stay; length of stay coupled with follow-up may enable determination of reasons for premature departure and solutions to minimize early departure not related to positive outcomes. Second, obtaining length of stay data on houses across the country may enable stakeholders invested in the provision of SUD services to determine the extent of RH utilization (i.e., rates of initiation, engagement, and retention with RH). Collection of basic demographic data provides the opportunity to evaluate access and equity of RH services by individuals representing various population groups and the health-related social needs of individuals served by the RH in varying geographies (rural and non-rural).

#### Proposed Measures

- Demographics (Age, gender, and race and ethnicity).
- Familial (Marital status and children under the age of 18).
- Primary SUD type.
- Referral Source.
- Length of Stay [entry date and exit date].

Collection Timepoints: Intake and exit.

### Tier 2: Light

This tier covers all measures in tier 1 as well as one measure of recovery capital, defined as the resources internal and external to an individual that can be drawn upon to initiate and sustain recovery (Granfield & Cloud, 1999). By measuring recovery capital though a validated scale such as the Brief Assessment of Recovery Capital (BARC-10), the original 50-item Assessment of Recovery Capital (ARC), or the 23-item Multidimensional Inventory of Recovery Capital (MIRC), RH providers can use a single measure to help identify areas that may need additional support. A measure of recovery capital is also collected at intake along with the other measures collected in tier 1 and at departure.

#### Proposed Measures

- All minimum measures.
- Recovery Capital: 10-item, Brief Assessment of Recovery Capital (BARC-10) (Vilsaint et al., 2017), 50-item, Assessment of Recovery Capital (ARC) (Groshkova et al., 2013), 23-item, Multidimensional Inventory of Recovery Capital (MIRC) (Bowen et al., 2023).
- Physical and mental disabilities and co-morbidities.

Collection Timepoints: Intake and exit.

### Tier 3: Moderate

This tier covers all measures in tiers 1 and 2 as well as additional measures that assess factors that may impact development of recovery capital and residential retention. These factors such as intrinsic factors (e.g., mental health, craving, trauma, etc.) and non-specific environmental factors (e.g., therapeutic alliance) have all been found to impact an individuals’ engagement with recovery care (Boddapati et al., 2014; Dass-Brailsford & Myrick, 2010; Fatseas et al., 2018; Rübig et al., 2021). Assessing these factors periodically (i.e., intake, 1 month, and every three months until exit), may help staff ensure that residents’ needs are being met. For example, if a resident completes an alliance survey at one month and the score indicates low alliance, a conversation can occur to determine steps that can be taken to support the resident to help them feel more in alignment with house staff and peers. Further, for the trauma measures listed, if a resident’s score indicates they have experienced trauma, a trauma-informed care approach should be provided for the resident (Substance Abuse and Mental Health Services Administration, 2014). For this tier, it is recommended that at least one measure at minimum is collected but more may be added as capacity allows.

#### Proposed Measures

- All light measures.
- Mental health brief screeners: 4 and 8-item, Patient Health Questionnaires (PHQ-4) and PHQ-8 and 7-item, Generalized Anxiety Disorder (GAD-7) (Levis et al., 2020; Löwe et al., 2010; Spitzer et al., 2006).
- Therapeutic alliance: 12-item, Fletcher Recovery Housing Alliance Measure (FRHAM-12) (Johnson et al., 2023).
- Trauma: Adverse Childhood Experiences (ACEs) Questionnaire (Felitti et al., 1998), Brief Trauma Questionnaire (BTQ) (Schnurr et al., n.d.)
- Quality of Life: Centers for Disease Control and Prevention 1-item, Quality of Life (Dumas et al., 2020).
- Craving/Desire to Use: 2-item SUD Continued Use (Rollnick et al., 2008).
- Locus of Control: 4-item, Locus of Control (Wang & Su, 2013).

Collection time points: Intake, 1 month, every three months until exit, exit.

### Tier 4: Robust

This tier covers all measures listed in tiers one through four as well as measures collected 1–12 months post-recovery home stay. This tier is a comprehensive approach and includes measures that may enable determination of the long-term impacts of RH programs and services on factors that have known implications on long-term recovery such as employment, housing, legal involvement, and healthcare and recovery support service engagement. In addition to these measures, a RH program may consider adoption of the robust, REC-CAP platform, an evidence-based assessment and recovery planning instrument.

#### Proposed Measures

- All moderate measures.
- Substance Use: 10-item, Alcohol screening questionnaire (AUDIT), 2-item, Drug Abuse Screening Test (DAST-2) (Johnson et al., 2013; Tiet et al., 2017).
- Employment status.
- Housing status.
- Legal involvement.
- Social connections/relationships/family.
- Health care services utilization and ongoing participation in recovery support services (chronic condition management).
- REC-CAP System (Best, n.d.)

Collection timepoints: Intake, 1 month, every three months until exit, 1 month follow-up, every three to six months follow-up out to two to five years. If implementing, REC-CAP, data collection specifications are directed by the Advanced Recovery Management Systems (ARMS) team.

The RH care continuity framework provided for assessment of initiation, engagement, and retention with RH along with example outcome measures for tiers I-IV enables the development of a quality matrix for which a minimum set of measures for RH can be systematically collected (Table 2). This model provides flexibility as well as allowing for innovation in evolving documentation of resident and program outcomes that that emerge as important from the perspective of RH operators or in the RH research.

**Table 2:** Recovery housing tiered quality measures matrix, 2023

Recovery House- Level	Resident-Level	Process Measure		Outcome Measure		Collection Time Points			
Degree of Outcomes Monitoring/ Quality Measures	Resident Characteristics • Age • Gender • Race • Ethnicity • Referral Source	Length of Stay	Healthcare Access • Mental health • Physical health • SUD Care • ED/Inpatient services	Health & Well-Being	Social • Interpersonal • Employment • Criminal Justice	Intake	During Stay	Exit	Follow-Up
Tier I	X	X				X		X	
Tier II	X	X			X	X		X	
Tier III	X	X		X	X	X	X	X	
Tier IV	X	X	X	X	X	X	X	X	X

ED, emergency department

## Conclusion

As the service capacity of RH continues to evolve, it is imperative that RH providers systematically adopt a universal set of data collection measures. A standardized set of measures for SUD recovery support services are needed to systematically assess the effectiveness of all services and supports within the SUD continuum of care for individuals of varying races, ethnicities, genders, and within various geographic locations (rural and non-rural). A set of quality measures supporting evaluation specific to RH programs are proposed, but these measures can translate to other care entities providing recovery support such as recovery community organizations and other settings. Employing a universal set of brief measures across recovery care settings that offers flexibility and utility for operators will also enable Federal agencies like CMS and others invested in public health to obtain the evidence required to determine the effectiveness of RH and other recovery support servicesand ultimately identify SUD services that improve health equity, affordability, and leverage partnerships that achieve the healthcare system transformation needed to support quality comprehensive SUD treatment and recovery care. The evidence for RH, with its peer support centered element is promising, but the privately owned and operated service delivery model of RH varies significantly due to the limited oversight, lending to lack of systematic universal data collection and consequent evidence. A set of tiered data collection sets with brief validated instruments are provided to support RH of all levels in building needed evidence.
